# Fuzzy Ontology and LSTM-Based Text Mining: A Transportation Network Monitoring System for Assisting Travel

**DOI:** 10.3390/s19020234

**Published:** 2019-01-09

**Authors:** Farman Ali, Shaker El-Sappagh, Daehan Kwak

**Affiliations:** 1 Department of Information and Communication Engineering, Inha University, Incheon 22212, Korea; farmankanju@gmail.com (F.A.); shaker_elsapagh@yahoo.com (S.E.-S.); 2 Department of Information Systems, Benha University, Banha 13518, Egypt; 3 Department of Computer Science, Kean University, Union, NJ 07083, USA

**Keywords:** intelligent transportation system, feature extraction, social network, sentiment classification

## Abstract

Intelligent Transportation Systems (ITSs) utilize a sensor network-based system to gather and interpret traffic information. In addition, mobility users utilize mobile applications to collect transport information for safe traveling. However, these types of information are not sufficient to examine all aspects of the transportation networks. Therefore, both ITSs and mobility users need a smart approach and social media data, which can help ITSs examine transport services, support traffic and control management, and help mobility users travel safely. People utilize social networks to share their thoughts and opinions regarding transportation, which are useful for ITSs and travelers. However, user-generated text on social media is short in length, unstructured, and covers a broad range of dynamic topics. The application of recent Machine Learning (ML) approach is inefficient for extracting relevant features from unstructured data, detecting word polarity of features, and classifying the sentiment of features correctly. In addition, ML classifiers consistently miss the semantic feature of the word meaning. A novel fuzzy ontology-based semantic knowledge with Word2vec model is proposed to improve the task of transportation features extraction and text classification using the Bi-directional Long Short-Term Memory (Bi-LSTM) approach. The proposed fuzzy ontology describes semantic knowledge about entities and features and their relation in the transportation domain. Fuzzy ontology and smart methodology are developed in Web Ontology Language and Java, respectively. By utilizing word embedding with fuzzy ontology as a representation of text, Bi-LSTM shows satisfactory improvement in both the extraction of features and the classification of the unstructured text of social media.

## 1. Introduction

Transportation information collection from social networks and its utilization for travel safety are two challenging issues in Intelligent Transportation Systems (ITSs). Traffic networks can be monitored by sensor devices and social network data. At present, ITSs utilize sensor devices to monitor all aspects of transportation networks. The ITS office uses these devices to gather data about roads and the speed and position of vehicles, etc. After analyzing these data, the ITS office shares public safety notifications regarding traffic or hazards with travelers or notifies the highway department regarding road conditions. However, ITS may not be able to collect precise traffic information from these sensors. In addition, travelers need to use smartphones for the collection of transport data, which help them travel safely. Therefore, both ITS and travelers need smart methodology and social network data. ITSs can employ social network data to support traffic and control management and examine transport services. While travelers can use these data to identify a risk zone before entering it, which can save time and fuel consumption. The analysis results of these data can help travelers travel safely, solve the traffic jam-related problems, and enhance traveling in urban areas. However, in social media, information related to traffic such as car accident and traffic jams comes with unexpected text, which makes the task of transport text mining more difficult for ITSs [1,2,3].

The analysis of social media data regarding transportation features is another problem in the natural language processing domain. Several systems have been proposed to handle these unstructured data [4,5]. Sentiment analysis is well-established to study the unstructured text of social media and to understand the posted thoughts of users. It determines subjectivity and polarity in a user-generated text. The Named Entity or the aspect in sentences are subjective, such as the name of the person, location, department, and the vehicle type. Various extraction approaches are employed to detect these entities and identify the polarity of them by using sentimental words in sentences. However, researchers have been proposing different approaches for sentiment analysis in the transportation domain [6,7,8]. These types of approaches only focused on to discover sentence or document level opinions. These types of approaches may not satisfy the need of ITSs and travelers as per their expectations. ITSs and travelers need more meaningful information from social media text in terms of specific aspects or features of transportation. For example, ITS may need to understand the thoughts of people regarding the ‘city road’ in order to improve their conditions, and travelers may be interested to know about the ‘street,’ ‘traffic jams,’ or the ‘traffic facility’ of the city. In addition, sentiments about these features in the user-generated text are expressed in different ways. Therefore, it is important to detect sentiments with respect to the features or aspects before identifying sentence polarity.

Researchers have developed different techniques to influence machine learning methods. A standardized learning method called *bag-of-words* is utilized to represent sentences by the words they comprise. However, this method neglects word order. An *n-grams* method is used to represent sentences by the sequence of words. *Terms frequency-inverse document frequency* (*tf-idf*) is employed to represent sentences by the weight of words. *Latent Dirichlet allocation* (*LDA*) discovers the topic for each document. However, LDA produces noisy topics, neglects the relationship between the topic and documents, and generates topics that contain irrelevant words. These methods are unable to illustrate the semantic meaning of each word in sentences. In addition, word embedding is utilized to train machine learning algorithms, which learn to identify the relationships between features. Word embedding misses the fundamental semantics of the features and entities according to a specific domain. Ontologies are the best method that provides a semantic knowledge of concepts and their relationship in a specific domain.

In this paper, a fuzzy ontology-based semantic knowledge with word embedding is proposed to enhance the task of transportation features extraction and sentiment classification using the Bi-directional Long Short-Term Memory (Bi-LSTM) algorithm. This semantic knowledge describes concepts and their relationships in the transportation domain and discovers the most relevant features in social media text. The word embedding model is trained using datasets of different social media and experiments are conducted with Support Vector Machine (SVM), Convolutional Neural Network (CNN), Recurrent Neural Network (RNN), and Bi-LSTM. The result shows satisfactory improvement in the accuracy of sentiment classification. The main contributions of this research are the following:

- A natural language processing method is developed that identifies the user-generated text regarding transportation features on social media to understand transport facilities and issues.
- Word2vec model called skip-gram is utilized to represent each word in the user-generated text with a semantic meaning.
- A fuzzy ontology-based semantic knowledge is developed to improve deep learning techniques that extract more accurate aspects or features from the transportation text.
- A novel fuzzy ontology and word embedding is used as the source of data to train the Bi-LSTM model for the classification of unstructured text on social media.

This paper is organized as follows: Section 2 presents the related work in ITS, sentiment classification, ontology, and deep learning. Section 3 presents the architecture and internal process of the proposed system. Section 4 presents the experimental results. Section 5 presents the findings and limitations of our model. Finally, Section 6 presents the conclusions.

## 2. Related Work

Traffic information extraction from social media contents and processing transportation data are interesting issues in the intelligent transportation system (ITSs). Transportation networks can be monitored in two distinctive ways: sensor device-based transportation monitoring and social network-based transportation monitoring. Currently, ITSs use sensor devices to monitor all aspects of transportation networks. In sensor-based monitoring, the ITS office gathers information about the road and the speed and position of vehicles. However, sensor-based monitoring systems may not able to gather accurate traffic information. Also, travelers use smartphones to gather data about transportation, which help them to travel securely. Sensors devices have limitations and are incapable of collecting suitable traffic data. Therefore, a smart methodology is required to extract and interpret traffic information and deliver useful data automatically. Such a methodology can enhance the protection of transportation, solve the problems related to traffic jams in mega-cities, and improve traveling in urban areas. In social network-based monitoring, the analysis of a massive amount of information related to traffic on social network platforms has made the task of transportation sentiment analysis more difficult for ITSs. A variety of analyses has been performed to handle these challenges, and several systems have been proposed for transport network issues [1,2,3].

A vehicular cloud service for route planning is developed where travelers share traffic images by utilizing the vehicles’ on-board cameras. In this existing system, a collaborative traffic image-sharing system called Social Vehicle Navigation (SVN) allows drivers in the vehicular cloud to report and share visual traffic information where this information is filtered, refined, and condensed into a traffic digest. These digests can provide more reliable information about the road situation to support the users’ route decision making [9]. A cloud-based service system is developed to protect the ITS’s energy [10]. In this system, transportation information is stored in a storage facility. This facility avoided the utilization of hard disks at a user site and enhanced responsibility. Real-time traffic conditions are recognized by mobile vision approaches in the existing system. The road-side camera data are collected and utilized to build an efficient route of navigation, which helps save time and fuel consumption. Transportation modeling challenges have been discussed in various approaches [11]. One approach used deductive data analyses and intelligent algorithms to infer useful patterns from big transportation data. These patterns enhance the accuracy and flexibility, and handle uncertain problems found in big data. A leapfrog technique-based system is utilized to enhance the performance of the map-matching algorithm in transport data centers [12]. This existing system utilizes travel paths within reserved organizations and map-matching in public in order to manage location data according to the specification of data privacy. A fuzzy logic and genetic algorithms based on the NeverStop system is proposed to handle the issues of big data in ITSs [13]. This framework utilizes devices based on radio frequency identification (RFID) in order to collect data about those vehicles which wait for the red traffic signal and pass the green signal. After this information retrieval, EBOX II devices in the main servers are used to retain the information for further analysis. A system based on hierarchical cloud architecture is presented for vehicular networks [14]. This system utilizes different resources in ITS organization such as ITS data centers and vehicles to design a universal cloud atmosphere for transportable vehicles. This system allows cloud services for vehicles to provide a communication facility with high quality.

Extracting valuable data from social media in the field of text mining is a challenging task. Recently, several techniques have been proposed in order to handle issues in sentiment classification and information extraction such as fuzzy logic, semantic knowledge, deep learning, and word embedding [15,16,17,18]. Currently, the extraction of traffic information from social media and its utilization for traffic activity have become topics in ITS [1,5,11,19,20,21,22]. People use social media to share their thoughts about various challenges in transportation and ITS services (e.g., accidents, landslides, and traffic jams), and other users respond to the same issues with emoticons or text. However, the users’ thoughts regarding transportation are expressed in terms of aspects such as “*Bus drivers are helpful in New York Downtown*” [1]. Both systems and users have faced difficulty to understand the expression of people’s thoughts. Further, the conversion of data into useful information may be valuable for transport services, and traffic control management systems. However, to handle transportation-related issues and extract meaningful data, it is necessary to remove useless information and then identify the transport features and events.

Sentiment classification has been discussed since the early 2000s, and researchers have been proposing systems to analyze people’s thoughts and emotions from social network contents [23,24,25]. Recently, people have become fascinated with sharing and discussing their thoughts about hot topics on social media, such as TripAdvisor [26,27], Twitter [18,28], and Facebook [29,30]. Two methodologies are utilized to analyze these data, including lexicon-based approaches and machine learning approaches. Lexicon-based approaches are based on the word dictionary with positive and negative scores. SentiWordNet is one of the most widely used lexica in the field of sentiment classification [31,32]. However, due to the different interpretations of words, the lexicon-based approach may not achieve good results. To solve this issue, transportation-dependent lexicons are presented for the proposed system. On the other hand, machine learning approaches such as the multilayer perceptron (MLP), logistic regression, support vector machine (SVM), and naive Bayes (NB) models require a training dataset to learn the model from corpus data, as well as a testing dataset to verify the performance of the resulting model [22,25,28,33,34].

An ontology-based system is presented to enhance the accuracy of topic modeling [32]. In this existing system, the authors employed ontology to detect suitable features or entities after clustering. The result of using this ontology-based system showed the accuracy of features detection was highly enhanced. Feature-level sentiment classification based on ontology is presented to define the semantic relationship among concepts in a specific domain [27]. This existing ontology reduced the effort otherwise required to implement an expert system. An ontology-based system was developed for the management of traffic accidents [35]. This existing ontology comprises useful information about pedestrians, climate, environments, and roads that are employed for traffic safety.

While smart transport management systems are usually established according to the needs, fuzzy logic-based ontology is very limited to expressing and retrieving information. Two different systems of transportation sentiment analysis were developed using fuzzy ontology [1,3]. In the first system, the polarity of 6 different aspects is calculated to monitor transportation services. However, some of these aspects may neither help monitor all transport activities nor help ITSs. In the second system, the study authors presented another fuzzy ontology-based sentiment classification system to solve the above-mentioned problems. This existing system helps ITSs and provides a facility during traffic monitoring.

Bi-LSTM and RNN-based natural language processing are developed to recognize the safety issues with respect to e-cigarettes [36]. In this existing system, authors used a testbed, which collected e-cigarette information from the world’s largest online forum. The authors trained the word embedding model to represent the semantic meaning in the online discussion data and developed a Bi-LSTM model to detect safety problems from unstructured information. The ontology and LSTM-based system is presented to handle construction project information [4]. This existing system employs the big data of construction projects as a source of data for the business information management system. This system presented that LSTM and RNN with ontology can accurately predict the quality problems in projects. The ontology and LSTM-based system was developed for topic modeling [37]. This system presented the challenges of ontology-based topic modeling and statistical approach-based topic modeling. A Bi-LSTM and Gated Recurrent Unit (GRU), along with word embedding, is utilized for sequence learning [38]. The result of this existing system shows that Bi-LSTM improves results as compared with the traditional baseline for classification. The system also shows the improvement of Bi-LSTM in the semantic representation of the online user-generated content.

Traditional approaches have primarily been used in previous research work for text mining and sentiment analysis, which can relieve the problem to some extent. However, a method for transportation data analysis is proposed, which is based on Bi-LSTM, Word2vec, and fuzzy ontology. The existing transportation data analysis approaches are complex to implement, and may not extract the real semantic meaning of words. In addition, crisp ontology may not be sufficient for the transport domain. Therefore, fuzzy domain ontology is developed and Bi-LSTM explores the information from this model in order to improve the tasks of transportation feature extraction and text classification.

## 3. Fuzzy Ontology and LSTM-Based Text Mining

**Figure 1:**
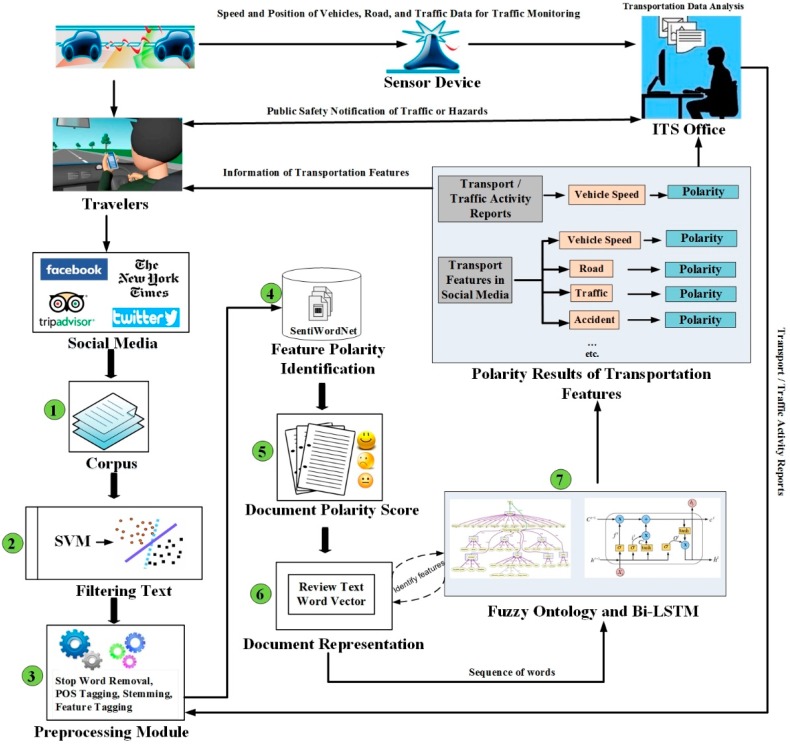
Proposed system architecture.

This section presents the framework of the proposed transportation network monitoring system, which is based on fuzzy ontology and Bi-LSTM. Figure 1 presents the complete architecture of the proposed system. It supports a series of text analysis functions, including information retrieval, data filtering, polarity detection, sentiment analysis, and information extraction. The proposed framework is divided into four modules. Each module contains different parts as follows:

- Data collections, transportation data filtration using SVM, and preprocessing of text.
- Feature polarity of identification and document polarity score.
- Word embedding for document representation and fuzzy ontology-based semantic knowledge for feature extraction.
- Bi-LSTM-based sentiment classification.

Data collection about specific feature is a challenging task in the sentiment analysis domain. Various queries are generated and different APIs are used to collect the most relevant information regarding transportation features. The proposed system collects the user-generated text related to transportation from ITS office reports and social media platforms such as Facebook, Twitter, and news articles (Task 1 in Figure 1). After data is collected, the precision is recognized as very low because of noise data. The noise data can make the feature extraction task more difficult and affect the polarity classification results. Therefore, Support Vector Machine (SVM) is employed to remove unrelated text (Task 2 in Figure 1). SVM classifies the data into two classes: positive and negative. Only the positive class text of the SVM classifier is considered as transportation text and the negative class text is removed. Social media data contains special symbols and other valueless content. Therefore, the user-generated texts are preprocessed to remove stop words, tokenize the texts, and part-of-speech tagging is applied to the texts (Task 3 in Figure 1). In the second module, features are extracted along with opinionated words using the n-gram approach. The n-gram method extracts features efficiently but cannot indicate the overall opinion. Therefore, SentiWordNet is employed to identify the polarity of the feature (Task 4 in Figure 1) Furthermore, the average polarity score of features in each sentence is computed and considered as a sentence polarity score. Then, the sentiment labels are assigned to each individual sentence in the document (Task 5 in Figure 1). The word embedding approach called Word2vec is used to represent the text in the document with low-dimension. The skip-gram model of Word2vec, which represents words as real-value vectors is applied (Task 6 in Figure 1). In the last module, the proposed system explores the domain knowledge from fuzzy ontology to improve the task of transportation features extraction using ML algorithms. The Bi-LSTM model, which conducts an accurate sentiment analysis of the transportation features and events, is constructed (Task 7 in Figure 1). A detailed description of each module is presented in the following sections.

### 3.1. Data Collection

This section provided the details of the dataset. Our research data related to transportation are collected from two different sources: ITS office reports and social network platforms. The dataset consists of 500,000 sentences. The data collection steps are explained below:

*Data collection from ITS Reports:* The online transportation reports contain multiple sentences, which are segmented into sentences. Keyword matching mechanisms are applied, and sentences are collected, which are related to 7 different features of transportation.

*Data collection from news articles:* The New York Times Developer Application Programming Interface (API) (https://developer.nytimes.com/) is used to retrieve news articles. These articles contain transportation-related information published between April 2017 and July 2017. The collected data contains irrelevant and unstructured text. Therefore, irrelevant data are removed and only those texts that are written in English are detected [39]. The lengthy news articles are split into paragraphs and then a keyword-based search technique is applied to determine the most relevant information.

*Data collection from TripAdvisor:* TripAdvisor data are retrieved, which are related to three different entities, including the city name, city features, and the location (e.g., hospitals, train stations, parks, bus station, bridges, hotels, and restaurants). The city name along with city features are employed as search queries to retrieve the user-generated text with the metadata. The retrieved dataset from TripAdvisor contains text about London and New York and the abovementioned city features [1]. The average length of each sentence is 80 words.

*Data collection from Facebook:* The Graph API along with the Java client (RestFB) is utilized to gather data from Facebook pages [30,40]. Specific pages are selected that contain transportation data about London and New York such as the transport of London, the New York State Department of Transportation and future transportation. All posts from these pages are collected that were published between March 2017 and January 2018. Then, all the user comments and responses (emotions and reactions) are gathered about these posts.

*Data Collection from Twitter:* The Twitter APIs, such as REST APIs and Streaming APIs, were employed to collect tweets comprising transportation information. The REST APIs permit users to utilize queries for the most recent tweets, while the Streaming API permits users to gather Twitter contents within a specified time period [1]. Our constructed queries were based on keywords, radius, centroid, and operators (OR and AND) (e.g., Vehicle AND (Accident OR Collision)). 500 keywords related to transport are utilized for the construction of these queries. However, users are only allowed 350 queries per 15 min by REST APIs, and 3200 recent tweets are collected per query. The constructed keyword-based queries extracted tweets related to transportation features.

### 3.2. Transportation Data Filtering

**Figure 2:**
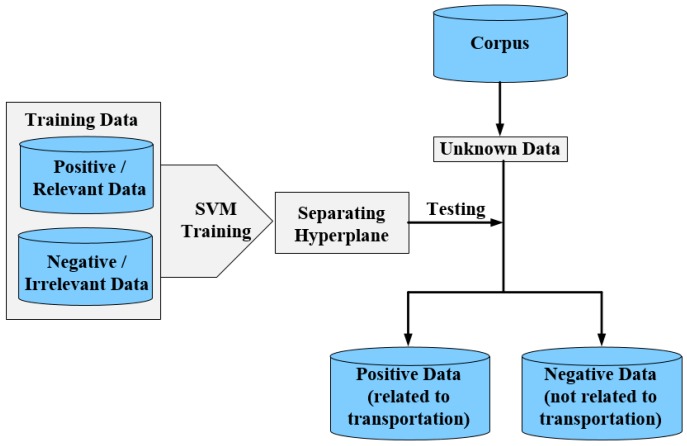
SVM-based data filtering.

In the sentiment analysis domain, various systems have been proposed to filter out noise data, such as a proximity function [41], multiple noise filtering system [42], and manual filtering [43,44]. However, preprocessing steps are required in proximity function-based filtering, which is a time consuming and complex task. In a multiple noise filtering system, each filter is assigned a specific task, such as a special corrector removal and misspelling detection. In manual filtering, experts must check each sentence in a document to identify the required text. However, it is a difficult task to evaluate each word by multiple filters or to manually check millions of sentences. Therefore, the SVM-based filtering system is utilized in our proposed system. The task of data filtering is directly related to the transportation text inquiry of the collection section. Since various queries are utilized to retrieve transportation related text, it is guaranteed that a large volume of irrelevant texts is collected in the corpus. The filtering module removes all contents to increase classification accuracy. SVM is applied to remove irrelevant text. It identifies the best possible hyperplane to distinguish useful text from the useless text. Chung proposed a renowned library called LIBSVM [33]. It classifies multi-classes employing two phases. In the first phase, SVM makes a model of the training data. In the second phase, SVM uses that model to obtain facts of the testing dataset. The SVM model for data filtering is presented in Figure 2. This SVM model is trained using the balanced dataset of relevant and irrelevant cases. After training, a decision boundary is achieved, which is the classifier for the collected data. This classifier categorizes the unknown data into positive and negative classes. The positive class data is considered the transportation data and the negative class data is considered irrelevant and filtered out. Let us consider the sentence “*Just saw a terrible accident on a road in Quezon*.” regarding the road and accident features. The n-gram technique is employed to extract these features. After extracting these features, a particular function is utilized to identify the value of the above sentence. The sentence is considered a transportation-related sentence if its value is larger than zero. Otherwise, it is considered negative and removed from the corpus. For example, the categorization procedure using SVM for the above sentence is: function (sentence) = 0.7 × Accident + 0.8 × Road + 0.3 × city name. The result is function (sentence) = 0.5 × 1 + 0.6 × 1 + 0.1 × 1 = 1.2. If the function (sentence) > 0, then it is defined as related to transportation (positive); In contrast, the sentence is declared as irrelevant text (negative) if the function (Sentence) <0.

### 3.3. Preprocessing

As the dataset is from TripAdvisor, Facebook, Twitter, and news articles, there must be discrepancies found in the text data, particularly if the text is from microblogs sites and social network sites. This module contains various steps to represent the dataset into a more structured form. These steps also facilitate the detection of features and its opinion-words extraction. The stop-word removal and cleaning are accomplished in the first step. This step filtered out URLs and words that occur regularly in the text. A well-known stop-word handler called ‘Rainbow’ is utilized to remove those contents such as symbols (@, date, #, etc.) and articles (a, an, the) which do not contribute to the sentiment. Next, part-of-speech (POS) tagging is applied. To allocate POS, the texts are split into sentences and Stanford CoreNLP was then applied. After POS tagging, the proposed system confirms that every sentence has a noun and a verb. Next step is tokenization, where a composite text is separated into small tokens. However, word spaces and delimiters generally occur in the composite text. Therefore, the n-gram tokenizer is employed to remove word space and delimiters. The outcome is then kept in the form of an array for further processing [45]. Next, the proposed system used a suffix-dropping algorithm for stemming and lemmatization. Stemming converts words to their root form, while lemmatization defines the lemma of the words used in the text. The lexical context is easily achieved from each word after lemmatization. For example, “accident” is related to “collision.” Therefore, the proposed system uses the stem and lemma words for further analysis. In the last step of the preprocessing module, the system changes characters and lowercasing. This step converts a sequence of characters that is repeated more than twice (e.g., “jammmmed” to “jammed”) to show words in the form of general words. Lowercasing transforms every word into lowercase to avoid confusion.

### 3.4. Feature Polarity Identification and Document Polarity Score

After data filtering and preprocessing, the SentiWordNet (SWN) tool is utilized to calculate the polarity of the features in each document and then applied the tool to whole document [46,47]. SWN contains 117,374 *synsets* with sentiment scores, which are annotated from *WordNet 2.0* [48]. A *synset* is a *WordNet* that contains synonym sets to represent a concept. It also comprises a grammatical class and a description gloss. In SWN, each *synset* is linked with three types of scores: Positive, Negative, and Objective. These scores display the relationship between the *synset* and its content in the form of a degree [47]. These scores range from 0 to 1, and the total of all three scores is equal to 1. To compute a polarity score for words and sentences, this system first passes a sentence through the preprocessing steps, which assign the POS to each word. The POS confirms the property of the word. Therefore, it is utilized to find the polarity score of the word. After POS tagging, not all of the senses of the words are taken into account. That is, only noun, verb, adjective, and adverb are searched in SWN. The proposed approach allocated zero scores to the opinioned word if the SWN did not contain a score for the sentiment word. When several sense terms are acknowledged in SWN about a particular term, then the arithmetic mean is calculated using the following equations [49]:

(1) $$Pol_{score}\;{\left(word\right)}_{pos}=\frac{\sum_{i=1}^{n}pol_{score_{pos_{\left(i\right)}}}}{n_{set}}$$

(2) $$Pol_{score}\;{\left(word\right)}_{neg}=\;\frac{\sum_{i=1}^{n}pol_{score_{neg_{\left(i\right)}}}}{n_{set}}$$

(3) $$Pol_{score}\;{\left(word\right)}_{obj}=\frac{\sum_{i=1}^{n}pol_{score_{obj_{\left(i\right)}}}}{n_{set}}$$

where “*Pol*,” “*pos*,” “*neg*,” and “*obj*,” show “polarity,” “positive,” “negative,” and “objective,” respectively. In addition, $n_{set}$ presents the total number of *synsets* of the word. After calculating the arithmetic mean for specific *synsets* of the word, the system obtained three scores: positive, negative, and objective. A detailed example of the polarity score calculation is provided to more clearly understand this step.

Let us consider the sentences in Table 1 which pertains to accident and road features. The word “terrible” is the sentiment word in the first sentence that has various entries in SWN. The word “terrible” is an adjective. SWN comprises four senses for the word “terrible” along with a positive and negative score as follows:

Terrible#1 (adjective), PosScore = 0, NegScore = 0.625
Terrible#2 (adjective), PosScore = 0, NegScore = 0.875
Terrible#3 (adjective), PosScore = 0, NegScore = 0.875
Terrible#4 (adjective), PosScore = 0.125, NegScore = 0.25

The combined positive and negative scores for the word “terrible” is identified using Equations (1) and (2), which are PosScore = 0.031 and NegScore = 0.656. The negative scores for each term are subtracted from the positive score as shown in the following equation:

(4) $$Score\;\left(word_{sense}\right)=\left[\mathrm{PosScore}\right]-\left[\mathrm{NegScore}\right]$$

The final score for the word “terrible” is computed by Equation (4), which is −0.625. Similarly, the same method is used for the opinion words “injured,” “closed,” “temporary,” “accident,” “crash,” “removed,” “start,” and “hopefully.” In column 3 of Table 1, the polarity scores of these words are presented. The final score for each sentence is then succeeded by computing the average values, as shown in Equation (5):

(5) $$ScoreFinal\;\left(sent\right)=\overset{n}{\underset{i=1}{\sum }}Score\;{\left(word_{sense}\right)}_{i}$$

The $Score\;{\left(word_{sense}\right)}_{i}$ in the above equation relates to the returned score by Equation (4). The final score of each sentence is presented in column 4 of Table 1. If the score is less than zero, then a sentence is negative. In contrast, the sentence is categorized a positive sentiment if the score is greater than zero. A sentence is categorized as neutral if the score is equal to zero. As the score of the first sentence is −1.06, which is less than zero, the sentence is classified as negative.

### 3.5. Word Embedding for Document Representation

**Figure 3:**
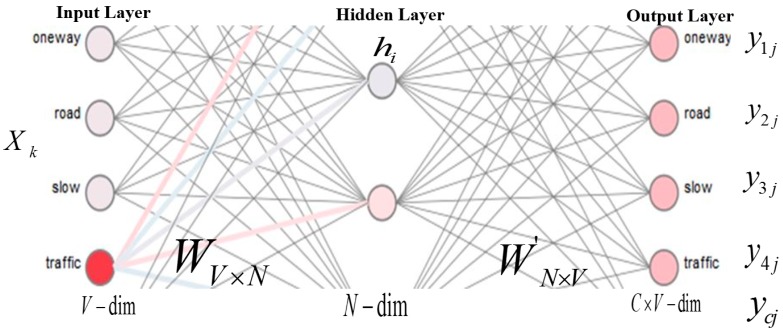
Trained skip-gram model for transport data.

Word embedding is a set of text processing approaches, where phrases or words from the dataset are plotted to vector values of real numbers. Two approaches, such as one hot representation and word embedding, are used to represent text data in the form of numerical data for advanced analysis [16]. However, word embedding has improved the performance of text mining tasks, such as sentiment analysis in recent research [13]. After labeling the dataset as discussed in the previous section, the Word2vec model is trained using the skip-gram approach on the large corpus of data. In the Word2vec model, a number is assigned to each unique word in the corpus. The training objective of this skip-gram model is to train a network that predicts the surrounding context words that occur in a given current word, as shown in Figure 3. In Figure 3, V, N, and C indicate the size of the vocabulary, the size of the hidden layer, and the number of words in the context, respectively. In this system, a word vector is passed through the hidden layer and the activation function that generates the probability distribution of words occurs in the original context of words. The description of this model is shown in Equation (6) [50]:

(6) $$\frac{1}{T}=\overset{T}{\underset{t=1}{\sum }}\underset{-c\leq j\leq c,j\neq 0}{\sum }\log p(w_{t+j}|w_{t})$$

In the above equation, *T* and *c* represent the number of unique words in the training dataset and the window size of the surrounding words, respectively. $w_{t}$ is the given word and $w_{t+j}$ are its surrounding words. Given a word $w_{t}$, the average log probability is maximized as shown in Equation (6). The result of this model is an array of semantic vectors and the semantic relationship between words, called word embedding. Word embedding helps predict the surrounding words of each word in the dataset. In the proposed system, a 200-dimensional word embedding model is applied to represent words. This model is composed of 200 words that are most likely to be the surrounding words of a given word.

Word2vec allows us to represent the transportation entities in the social network text and identify the semantic relationship between entities. For example, *jammed* is very similar to the context *congestion*. To improve the performance of word embedding, the top 10,000 words are trained and a unified symbol (LFW) is assigned to the remaining less-frequent words. A 200-dimensional word embedding model is generated after the training of transportation dataset.

### 3.6. Fuzzy Ontology-Based Semantic Knowledge for Transportation Feature Extraction

Word embedding is used to train machine learning algorithms that learn to identify the relationships between features. These ML algorithms sometimes miss the fundamental semantics of the feature according to their corresponding domain. However, the semantics of the individual domain are mostly available in the form of ontology. Ontology provides the semantic knowledge representation of concepts and their relationship in a specific domain. In this paper, the fuzzy ontology of transportation feature is considered where each class of it represents concept, and each property of it represents the relationship between concepts. The proposed ontology is used to influence the illustration of the word-level semantics in the process of feature polarity computation and word embedding with Bi-LSTM. The main task performed by the proposed ontology is discussed in detail below.

**Figure 4:**
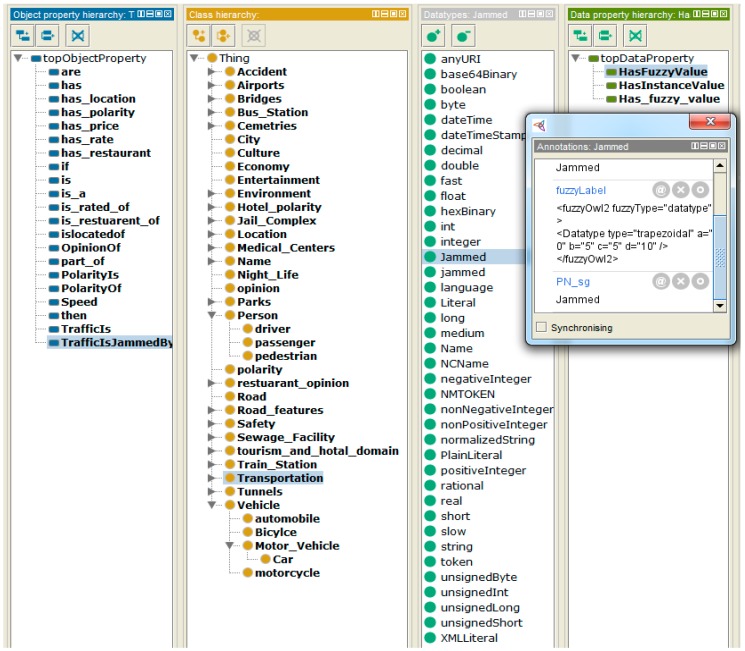
Ontology of transportation features.

The proposed fuzzy ontology offers information about the features and helps to perform accurate feature extraction and feature polarity computation. To advance the performance of feature extraction and polarity computation, a transportation ontology is developed that comprises expert knowledge to signify the semantic relationship among various concepts. It is significant to construct ontology manually, and subsequently approve the features of each concept. Otherwise, the constructed ontology would be vague and unusable. In addition, a classical ontology becomes very large, and difficult to utilize for feature extraction in a transportation domain. Therefore, first a classical ontology is built using Protégé-OWL to achieve productivity for the proposed ontology of transportation feature. A fuzzy OWL plugin is then utilized to transform the classical ontology into a fuzzy ontology as illustrated in Figure 4.

Our proposed ontology illustrates the concept of transportation features, such as roads, climate, vehicles, accidents, etc. It comprises a set of transportation-related entities and their relationships (e.g., “car” “is_a” feature of “vehicle”), which can be utilized to extract entities from the unstructured text of social media. The proposed ontology can also be utilized to categorize sentences efficiently, and to calculate the polarity of transportation features. Ali et al. presented the fuzzy concepts of transportation features in ontology [1]. A sentence “*Just saw a terrible accident on a road in Quezon*” is used from Table 1 to explain feature extraction in detail. Before feature or entity extraction, the preprocessing of text is performed to filtered out stop words, prepositions (on, in, of), and articles (the, a, an) from a sentence. It is essential to confirm that each sentence has a noun and a verb. Therefore, a sentence that has more than one conjunction is divided into two sentences to present a complete clause with one noun, one conjunction, and one verb. To extract the exact transportation features, each class of the ontology is considered as a feature and map it to the corresponding feature in a sentence. When a similarity is detected, the opinion words are extracted from the sentence and stored with the obtained transportation features. For example, “road,” and “accident,” are nouns in the above sentence. The keywords indicate that the sentence is about a road and accident feature. The opinion word for these features is “terrible,” which is used to find the polarity of features.

The proposed ontology and LSTM discover the domain information from fuzzy ontology in order to advance the task of transportation feature extraction employing ML algorithms. The deep learning classifiers are discussed in the next sections.

### 3.7. Bi-Directional Long Short-Term Memory (Bi-LSTM) Model

**Figure 5:**
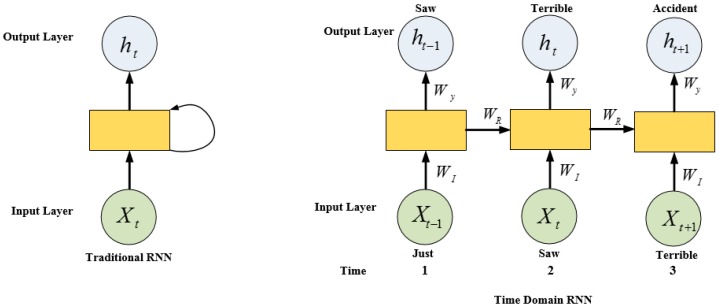
Traditional RNN and time domain RNN.

For decades, machine learning algorithms such as SVM and logistic regression have been used to classify the text at three levels: the aspect level, sentence level, and the document level. These models are shallow and trained on high-dimensional and sparse features, which may not solve the targeted problems in NLP [51]. Deep learning networks including the Multi-Layer Perceptron (MLP), Convolutional Neural Network (CNN), and Recurrent Neural Networks (RNNs) are used for the purpose of text generation, word representation, sentence modeling, and sentence classification. However, MLP is suitable for a small amount of data. The input of MLP is constant and related only to the current instant. It is unable to handle contextual information and variable sequence lengths to produce the correct results. MLP is time consuming and the classification results are worse than those of other models [52]. CNN and RNN have been compared for sentiment classification [53]. The authors reported that CNN provides better performance in extracting informative features whereas RNN is useful for modeling units in sequences. CNN uses the fixed size of a window which moves over a sentence to extract the local features from the sequence of words in a sentence. However, CNN is limited to extract the opinionated words in lengthy sentences and consequently miss the semantic meaning of words. Recurrent Neural Networks (RNNs) have shown great success in the task of sequence modeling, such as the time series, natural language processing, machine translation, and the dialogue system [50,54,55]. Much of the textual data in ITS systems are largely idle because of processing complications. The design of a predictive model for the excellence of RNN makes well-organized use of transportation-related text data. Traditional RNN consists of three layers: the input layer, the hidden layer, and the output layer. The functionality of the input and output layers of RNN and forward neural network is the same. However, there is no concept of time in traditional RNN. Therefore, the current word is only considered in the training process. The main feature of the distribution of an RNN architecture is to share information between time steps. Both traditional and time domain RNN models are shown in Figure 5. For example, the input time *t* = 1 is the token “just” for the input sequence “*Just saw terrible accident*” as presented in Figure 5. The forward propagation of RNN is described in the following equations [50]:

(7) $$h^{t}=f\;\left(w_{I}x^{t}+w_{R}h^{t-1}+b_{h}\right)$$

(8) $$h^{t}=f\;\left(w_{y}h^{t}+b_{y}\right)$$

Two outputs are calculated in the previous and current predictions at each time step. The previous and current predictions are calculated from Equations (7) and (8), respectively. The weights and biases are shared across the layer. Mostly the recurrence formula (tanh activation function) is used for f. In the output layer, the softmax activation function, which allows the output to be read as probabilities, is employed. However, RNN has no control over the increase of information in time and faces vanishing and exploding gradient problems. Therefore, several solutions including LSTM, exist to address these problems. Such solutions work well in the prediction of a large number of sequence data.

The main objective of this research is to identify the feature or entity types related to transportation events that can be considered as entity recognition tasks. In a social network platform, a user-generated tweet, comment, or post contains long text. Further, the words before and after the given word can be used to determine its semantic meaning. Therefore, a model is made that can handle the long text of a document and process texts both forward and backward. In this way, the system simultaneously captures the history and the forward information of the word. A Bi-LSTM language model is implemented to extract transportation features from the social media data.

**Figure 6:**
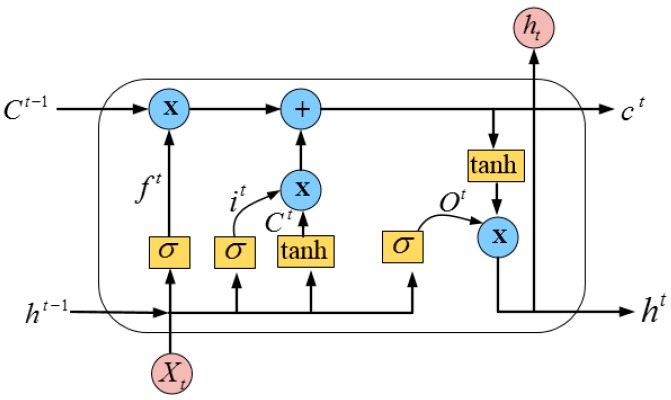
LSTM unit.

LSTM is one of the types of RNN architecture that shows extreme success in learning long-distance dependencies. The LSTM unit is shown in Figure 6 and it is specially designed as a memory cell to store previous information [56]. The unit of LSTM comprises a memory cell $C^{t}$, a hidden state $h^{t}$, an input gate $i^{t}$, a forget gate $f^{t}$, and an output gate $O^{t}$, which control the update and use of previous information. The output of the LSTM can be calculated by the following steps:

- First, the input data and the weight of the previous LSTM cell is required to be found. Therefore, according to the RNN formula, the memory cell value ${\bar{C}}^{t},$ $w_{xc}$, $w_{hc}$ are calculated at the current state as shown in Equation (9).
- It is important to control the flow of the current information on the memory cell state value. Thus, input gate $i^{t}$ value is computed to control the influence of current information as shown in Equation (10).
- The value of forget gate $f^{t}$ is computed to control the effect of historical data on the status value of memory cell as presented in Equation (11).
- After the calculation of the forget gate value, the status value of current memory cell $C^{t}$ is computed by using Equation (12).
- The output gate $O^{t}$ value is computed to control the status value of the memory cell by using Equation (13).
- Finally, the last LSTM unit output is calculated by Equation (14):
  (9) $${\bar{C}}^{t}=tanh\;\left(w_{xc}x^{t}+w_{hc}h^{t-1}+b_{c}\right).$$
  (10) $$i^{t}=\;\sigma \;\left(w_{xi}x^{t}+w_{hi}h^{t-1}+w_{Ci}C^{t-1}+b_{i}\right).$$
  (11) $$f^{t}=\;\sigma \;\left(w_{xf}x^{t}+w_{hf}h^{t-1}+w_{Cf}C^{t-1}+b_{f}\right).$$
  (12) $$C^{t}=f^{t}⊙C^{t-1}+i^{t}⊙{\bar{C}}^{t}.$$
  (13) $$O^{t}=\;\sigma \;\left(w_{xo}x^{t}+w_{ho}h^{t-1}+w_{Co}C^{t-1}+b_{o}\right).$$
  (14) $$h^{t}=O^{t}⊙tanh\;\left(C^{t}\right).$$

The final output of the LSTM is $h^{t}$, which is calculated by using the input feature matrix of the cell state and the feature matrix output of the output gate at the current time. The input data of the LSTM model is $x^{t}$. Its parameters are $w_{xc},w_{hc},w_{xi},b_{i},\;w_{xf},\;b_{f},\;w_{xo}$ and $b_{o}$. The output layer of the LSTM model is added with the softmax function to determine the probability outputs in (0, 1). This probability outputs show that the transportation feature contained in the social media text and ITS office reports may be positive or negative. Mathematically, the softmax activation function is defined in Equation (15):

(15) $${\mathrm{softmax}}^{kt}=\frac{\exp\left(x^{kt}\right)}{\sum_{\overset{´}{k}}^{c}\exp\left(x^{\overset{´}{kt}}\right)}$$

In the above equation, c and $x^{kt}$ represent the emotion category and the input of the time step *k*, respectively.

A series of text vectors and parameters are input to the LSTM model. These parameters are used in the network layers of LSTM to accomplish text feature learning, thereby updating and producing adequate results of sentiment classification.

**Figure 7:**
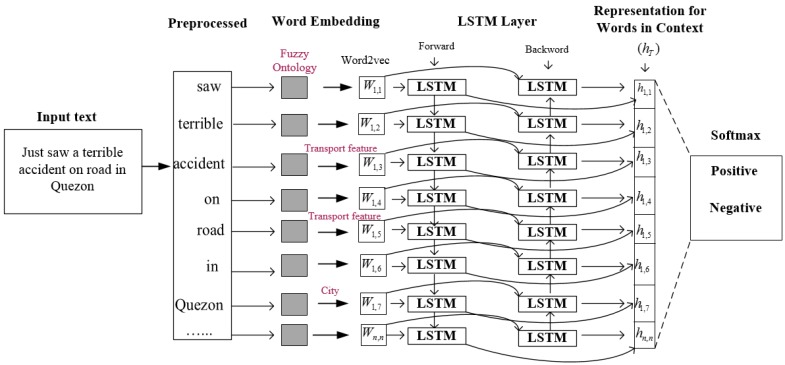
Word embedding and LSTM-based text classification.

The decision of transportation features polarity is also affected by the sequence of input context of features. In addition, single LSTM is limited to accessing only the past information and computing the output based on the learned information. For example, the input sentence ‘Saw a __accident’ is missing the word ‘terrible.’ LSTM utilizes only ‘Saw a’ to generate the missing word ‘terrible.’ However, based on the training data, the network may generate a new word, such as ‘small,’ ‘car,’ etc. Bi-LSTM has both past (‘saw a’) and future (‘terrible’) information, and can easily predict that the missing word is ‘terrible.’ Therefore, a Bi-LSTM is used to improve the determination of feature sentiment polarity. In the Bi-LSTM model, two LSTM neural network run in parallel. One network runs on the input sequence and other runs on the reverse of the input sequence. The procedure allows Bi-LSTM to capture both the history and future data. For example, when the vectors of text are assigned to Bi-LSTM, the first LSTM models the text at the start from left to right, and the other LSTM models the text at the end from right to left, and fully captures the information of features for sentiment analysis. The entire corpus is preprocessed in order to remove insignificant words. The fuzzy ontology of transportation is linked with word embedding to detect transport features and provide the semantic meaning of those words which are not in the domain (e.g., ‘Quezon’ is the name of a city). Then, a 200-dimensional word embedding model is trained to reduce complexity. This means that each word in the text is converted to a 200-dimensional semantic vector. Then, the embedding sequence words are passed to the Bi-LSTM layer. To avoid overfitting, 200 neurons are used in Bi-LSTM rather than employing a large size of the hidden layer. The output of the multi-layer LSTM network is then processed to the softmax layer, which predicts the sentiment label of the features in the input text. The graphical design of text classification using a fuzzy ontology, word embedding, Bi-LSTM, and the softmax layer is shown in Figure 7.

## 4. Experiments

In this section, a set of experiments was carried out in order to measure the usefulness of the proposed approach. The experiment results are provided regarding feature extraction, sentiment classification, different embedding dimensional vectors, and classification accuracy with different word embedding models. The dataset, which consists of 500,000 sentences related to transportation, was gathered from ITS office reports, news articles, and social network sites. First, the most discussed topics were extracted from ITS office reports. Then, those sentences were collected from news articles and social media contents with mention of the specific topics cited in the ITS office reports. In our experiment, the dataset contains information about 7 different features of transportation. These features are ‘Road,’ ‘Accident,’ ‘Vehicle,’ ‘Traffic,’ ‘Safety,’ ‘Location,’ and ‘Person.’ The detailed of the dataset was presented in Section 3.1 and Section 3.2. The sentences containing no target features were removed from the dataset. The dataset was automatically labeled using our proposed approach as presented in Section 3.4. The dataset was divided into two parts: 70% for training [including a development set (10%)] and 30% for testing. The proposed model with the existing ones is implemented using Java. The proposed system (fuzzy ontology and Word2vec with Bi-LSTM, as presented in Section 3.7) is compared with the three other models. The Word2vec model is trained on the transportation dataset and found that the optimal hyper-parameters for the word vector training to be 200 dimensions, the initial learning rate to be 0.5, the number of training epochs to be 15, and the vocabulary downsampling to be 1e^−2^. The LSTM two layers with 200 hidden vector dimensions is exercised for Bi-LSTM. A 0.5 dropout rate is assumed for regularization and the model is trained for up to 50 epochs. The optimal performance was from 30 epochs.

### 4.1. Experimental Settings

The protégé-OWL tool is employed to develop a fuzzy ontology and then fully implemented the respective processing steps along with SWN in Java. This proposed fuzzy ontology and SWN were used as a lexicon-based approach, and word embedding models were trained. In this work, four different sentiment classification approaches are utilized, including SVM, CNN, RNN, and LSTM. These ML classifiers were used with ontology, tf-idf, n-gram (6-gram language model), and LDA in order to present comprehensive evaluation results. Both RNN and LSTM are comprehensively discussed in Section 3.7. The details of other approaches are the following:

- *SVM.* LIBSVM with a linear kernel is suitable to classify the data [33]. SVM favors light training data so the training dataset comprised non-zero values. However, longer time was required during training. SVM is applied with a kernel-type radial basis function.
- *CNN.* Convolution Neural Network (CNN) has been used successfully for various text classification tasks [57]. Thus, CNN of the Waikato Environment for Knowledge Analysis (Weka) [58] library is configured along with a sigmoid activation function to implement a multi-layer network for the evaluation of the proposed approach.
- *Fuzzy ontology.* The ontology contains a set of transportation-related entities and their relationships as discussed in Section 3.6. It is used to extract the entities or features from unstructured text [59]. LSTM determines the domain information from fuzzy ontology in order to advance the task of transportation feature extraction.
- *tf-idf.* tf-idf is a standard and statistical model [60], which is used in the proposed work in order to understand how useful a feature or entity is to a document in the dataset.
- *LDA.* Latent Dirichlet allocation is a statistical model which generates abstract topics that occur in a large corpus of a dataset [61]. LDA is utilized to extract topics from transport-related data and compared the results with our proposed approach.

### 4.2. Performance Measure

A well-known method is conducted to evaluate the proposed system. Common metrics, such as precision, recall, function measure, and accuracy were used to evaluate the proposed methods. Mathematically, these measure metrics can be computed by the following equations [62]:

(16) $$\mathrm{Precision}=\frac{TP}{\left(TP+FP\right)}\;\times 100\%$$

(17) $$\mathrm{Recall}=\frac{TP}{\left(TP+TN\right)}\;\times 100\%$$

(18) $$\mathrm{Accuracy}\;=\frac{\left(TP+TN\right)}{\left(TP+FP+FN+TN\right)}$$

(19) $$Function\;Measure\;\left(FM\right)=\frac{2^{*}Precision^{*}Recall}{Precision\;+Recall}$$

where *TP*, *FP*, *FN*, and *TN* denote true positive, false positive, false negative, and true negative, respectively.

### 4.3. Evaluation Results

To highlight the usefulness of the proposed approach, the performance of the fuzzy ontology + Word2vec was compared with other approaches including ontology with tf-idf, n-gram, and LDA. In our experiment, the text corpus of seven different transportation features is formed for each method and computed the respective average precisions. The comparative results of the experimental standard models and our proposed model are shown in Table 2. Table 2 illustrates that fuzzy ontology with Word2vec (95%) has significant enhancement statistically over ontology with tf-idf (67.5%), n-gram (78.1), and LDA (82.5%). This high precision ensures that the proposed system can detect the most relevant transportation feature from the data corpus. The fuzzy ontology provides semantic knowledge to identify transport features in text and help Word2vec and LSTM to extract those terms that must be used in the task of sentiment classification. The Word2vec explores the transportation domain information from fuzzy ontology in order to improve the task of feature extraction. The obtained result shows that the proposed approach outperforms all of the other methods with a high margin.

The number of LSTM units and the number of neurons in the structure of the neural network are found to have an impact on the performance of the obtained results. LSTM with a small number of neurons may not be enough to classify complex data. In this work, the effect on the number of neurons with two LSTM layers was tested. The experiment results show that the Bi-LSTM neural network with 200 neurons achieved better accuracy among them in the classification task as in Table 3.

**Figure 8:**
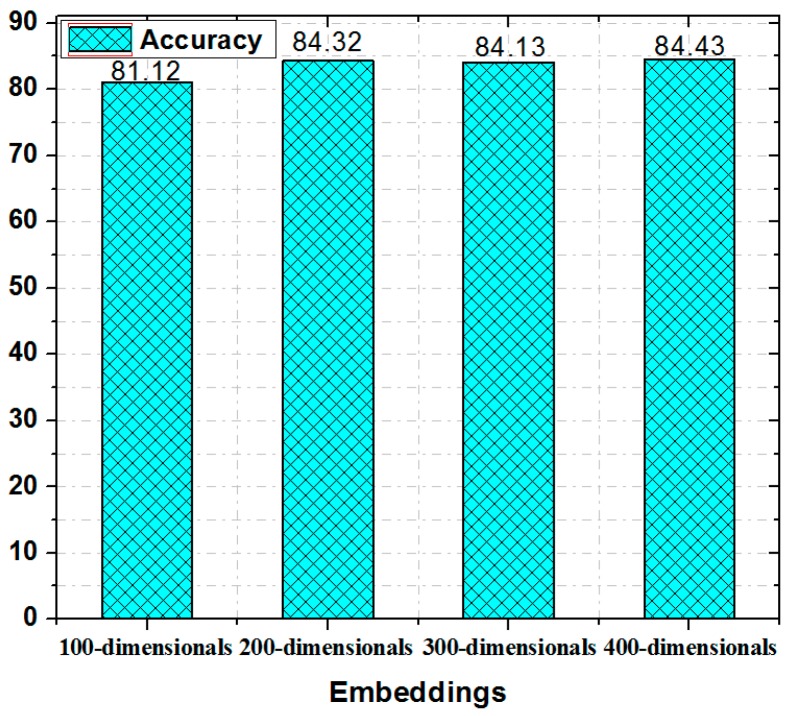
Accuracy of different embedding dimensional.

The performance of the proposed method is compared with the existing methods in Table 4. These results present the precision, recall, function measure, and accuracy of the different approaches. In case of tf-idf + SWN and n-gram + SWN, the obtained accuracy of SVM was 71% and 78%, respectively, which is higher than CNN and RNN. Furthermore, the accuracy of RNN was higher by 2% and 5% with respect to CNN in case of tf-idf + SWN and n-gram + SWN, respectively. With LDA + SWN, the measured accuracy of RNN was 76%, which is higher as compared to SVM and CNN. The best results were obtained while using SWN + Word2vec. Bi-LSTM outperformed SVM, CNN, and RNN in accuracy. With SWN + Word2vec, the accuracy of RNN was 80%, which is high by 8% and 9% as compared to CNN and SVM, respectively. However, the accuracy of RNN was lower by 4% than Bi-LSTM. The Bi-LSTM with Word2vec and fuzzy ontology clearly outperforms, word-level embeddings and sentiment classification as compared with other methods. The proposed method obtained precision, recall, function measure, and accuracy of 88%, 86%, 87%, and 84%, respectively. The most accurate results were achieved while using skip-gram vectors trained on 7 different transportation features. SVM with SWN and n-gram outperformed CNN with LDA and SVM with tf-idf in terms of all confusion matrix. However, the use of SVM with fuzzy ontology is complex and time consuming with a large dataset. Moreover, this method can miss the semantic relation between the feature and opinionated words, and requires extra steps during implementation. Fuzzy ontology-based semantic knowledge for transportation indeed make quality improvements for both Word2vec and Bi-LSTM. Based on the comparison results in Table 4, the improved performance of Word2vec with Bi-LSTM in the transportation text classification is better than those of previous systems. The Bi-LSTM has a memory function on the context and therefore it patterns the text in two directions that affect the decision of the sentiment polarity classification of features. The proposed system results are compared with those of the study by Ali et al. [1,3,8,27]. The precision and accuracy were found to be improved in the classification of the text related to transportation features. This shows that the combination of Bi-LSTM, Word2vec, and fuzzy ontology is effective for transportation sentiment classification. The Bi-LSTM model used has a long-term memory for the context of the text. However, traditional approaches neglect the context of the text, which results in inaccurate decisions with respect to polarity classification. Furthermore, the proposed system is analyzed with different sizes of word embedding vectors. Figure 8 shows that the results are not significantly improved when the size of the embedding vector is increased from 200 to 400.

**Figure 9:**
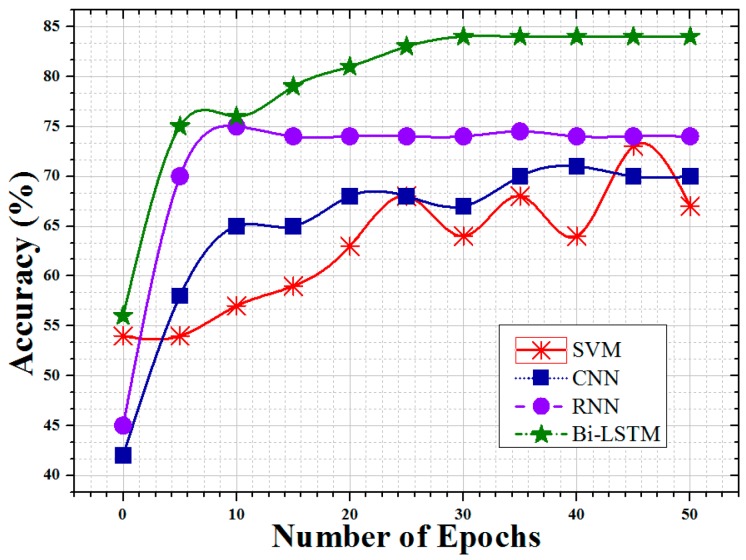
Classifier accuracy with different number of epochs.

The accuracy among SVM, CNN, RNN, and Bi-LSTM is compared in Figure 9 in terms of training epochs ranging from 0 to 50. This result indicates that ML classifiers can be successfully trained after 10 training epochs, which means that a proper number of training epochs is needed. The accuracy for all cases is shown to be stable after 20 epochs except of SVM case. The SVM’s accuracy is fluctuating beyond 20 epochs which is due to its sensitivity and varying weights after every training vector [63,64]. Overall, Bi-LSTM performs much better than others for all the ranges as shown in Figure 9.

**Figure 10:**
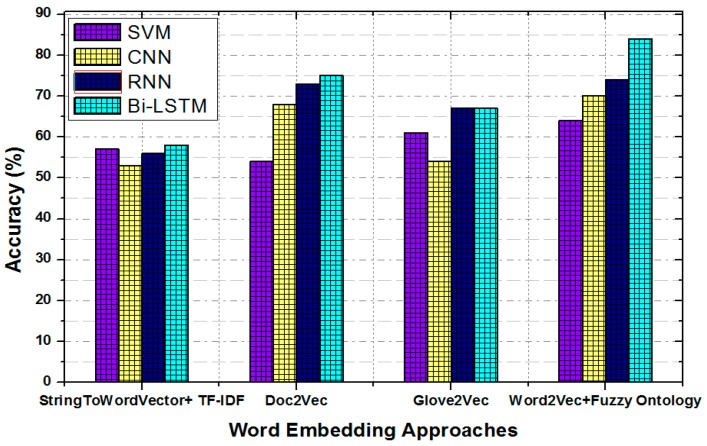
Classification accuracy of different baseline models with word embedding models.

Figure 10 shows the classification accuracy of different baseline models with the pre-trained word embedding models: StringToWordVector + TF-IDF, doc2vec, glov2vec, and Word2vec + fuzzy ontology. The obtained results indicate that ML classifiers perform well with the proposed pre-trained Word2vec + fuzzy ontology embedding model. This result also shows that the Bi-LSTM with the proposed word embedding model achieved the highest accuracy of 84% for sentiment classification. However, SVM, CNN, and RNN have obtained an accuracy of 64%, 70%, and 74%, respectively, which have worse accuracy performance as compared to Bi-LSTM. In addition, the accuracy of Bi-LSTM + Word2vec is higher by 26% with respect to Bi-LSTM + StringToWordVector, by 17% with respect to Bi-LSTM + Glove2vec, and by 9% with respect to Bi-LSTM + doc2vec. This shows that the fuzzy ontology with Word2vec and Bi-LSTM is more effective than ML algorithms with other word embedding approaches. In addition, embedding models and ML classifiers without ontology are unable to learn the features of the aspects. The reason is that many implicit aspects of transportation represent a different concept.

## 5. Discussion

### 5.1. Findings

The primary goal of our model was to present semantic knowledge to extract the most valuable information about transportation features from social media contents. Another aim of our model was to obtain useful knowledge that is not explained in the text: For example, providing semantic meaning to the word ‘Quezon’ as the name of a city. A fuzzy ontology with word embedding and Bi-LSTM is modeled, which works better than alternative models. Compared to feature extraction methods, our methodology achieved higher precision in terms of transportation features identification in social media contents. The existing LDA approach misses useful features during the generation of topics due to the limited dataset. The proposed approach solves the problem of targeted features extraction. In sentiment classification, the accuracy of the proposed system was 84%, which is the highest rate of accuracy as compared to other models. The reason is that Bi-LSTM with Word2vec discovers domain knowledge from fuzzy ontology and executes the semantic meaning of words. The improved accuracy of Bi-LSTM is attributed to its ability to retain previous information. Moreover, the search speed is enhanced as a result of the Bi-LSTM architecture, which refrains from searching a growing space with respect to the other models that explore comprehensive features and activities. The proposed Bi-LSTM model was evaluated with different sizes of word embedding vectors and worked well even with the limited training dataset.

### 5.2. Limitations

A number of limitations are noticed related to this study. There is a limitation of adequate labeled data sets regarding transportation features. Among the widely available data sets are the Google pre-trained word embedding models and the public online datasets. However, these pre-trained models contain information about domains other than transportation, which may not be appropriate for our specific purpose. The use of large-scale knowledge extracted from fuzzy ontology brings new challenges. The large number of concepts, their relationships, SWRL rule, and fuzzy instances in a fuzzy ontology may lead the semantic knowledge much complicated which makes the classification methods accordingly computationally complex. The current fuzzy ontology cannot handle sentences which are not associated with the aforementioned 7 features of our model. The main reason is that the incorporation of domain knowledge with machine learning algorithms may reduce the applicability of ML algorithms to other domains. Finally, the data from social media contents and ITS office reports are insufficient for transportation sentiment classification, and thus the integration of additional sensor data can improve transportation text classification.

## 6. Conclusions

In this paper, fuzzy ontology and word embedding as a text representation model was proposed to enhance the performance of transportation entities or features extraction using Bi-LTSM. Various sensible issues were studied, including concept assertion, features extraction, polarity determination, text representation, limitations of machine learning classifiers, and the role of fuzzy ontology in text classification using deep learning. The proposed system extracts transportation-related information from a large volume of social media datasets. Our proposed system offers a text classification method that automatically detects transportation entities in the large corpus and calculates the polarity of the entities. This system helps ITSs examine transportation services and supports traffic control and management systems. This system classifies unstructured data, enhances transportation facilities, decreases traffic congestion issues, and provides useful information in the form of features polarity in order to help travelers travel safely. This method can generate features from unstructured text and represent text with more semantic meaning in order to improve the performance of text mining and sentiment analysis. In this context, this method can be used to solve other issues such as entity detection, text mining, and sentiment classification.

In future research work, a fuzzy ontology-based semantic knowledge will be used to improve the performance of biomedical text classification tasks. The benefits of domain knowledge can be taken using machine learning classifiers to enhance the detection and classification of drug information.

## Figures and Tables

**Table 1:** Assigning polarity scores to words and sentences.

Example Sentence	Opinion Word	Word Polarity Score	Sentence Final Score	Sentiment
Just saw a **terrible accident** on a **road** in Quezon.	terrible	−0.625	−1.06	Negative
	accident	−0.437		
**Road** is **closed** due to **accident**, woman and three children **injured** after car **crash**.	closed	−0.47	−1.75	Negative
	injured	−0.62		
	crash	−0.23		
	accident	−0.437		
**Temporary traffic lights** on the West **road** have now been **removed**. **Hopefully** traffic will **start** to flow in the area	temporary	0.12	0.5	Positive
	hopefully	0.5		
	start	−0.12		

**Table 2:** The proposed system in comparison with other approaches in terms of feature extraction.

Transportation Features/Entities	Ontology + tf-idf	Ontology + n-gram	Ontology + LDA	Fuzzy Ontology + Word2vec
Road	61	81	85	96
Accident	65	85	83	96
Vehicle	70	78	90	98
Traffic	74	74	88	96
Safety	72	77	80	93
Location	64	79	77	93
Person	67	73	75	93

**Table 3:** Classification accuracy of Bi-LSTM with different number of neurons.

Neurons	Precision	Recall	Function Measure	Accuracy
50	72.1	71.9	71.8	71.8
100	76.3	76.0	76.0	76.0
150	82.5	82.0	82.0	82.0
200	88.0	86.0	87.0	84.0
250	83.6	83.2	83.2	83.2
300	83.3	83.7	84.0	83.0

**Table 4:** Sentiment classification performance of the different methods.

Methods	Precision	Recall	Function Measure	Accuracy
Ontology + tf-idf + SWN + SVM [3,27]	81	71	76	71
Fuzzy Ontology + tf-idf + SWN + CNN	65	64	63	64
Fuzzy Ontology + tf-idf + SWN + RNN	67	66	66	66
Fuzzy Ontology + n-gram + SWN + SVM [1,8]	84	80	82	78
Fuzzy Ontology + n-gram + SWN + CNN	66	62	60	62
Fuzzy Ontology + n-gram + SWN + RNN	70	67	66	67
Fuzzy Ontology + LDA + SWN + SVM	76	73	72	73
Fuzzy Ontology + LDA + SWN + CNN	77	74	75	75
Fuzzy Ontology + LDA + SWN + RNN	79	77	76	76
Fuzzy Ontology + SWN + Word2vec + SVM	72	74	73	71
Fuzzy Ontology + SWN + Word2vec + CNN	69	88	77	72
Fuzzy Ontology + SWN + Word2vec + RNN	80	82	81	80
Fuzzy Ontology + SWN + Word2vec + Bi-LSTM	88	86	87	84
